# 10-year trends in benzodiazepine, opioid, and concurrent use in hip and knee arthroplasty: a nationwide cohort study from the Netherlands

**DOI:** 10.2340/17453674.2025.44755

**Published:** 2025-10-10

**Authors:** Manuela YEPES-CALDERÓN, Rob G H H NELISSEN, Marcel L BOUVY, Frits L ROSENDAAL, Liza N VAN STEENBERGEN, Albert DAHAN, Maaike G J GADEMAN

**Affiliations:** 1 Department of Orthopaedics, Leiden University Medical Center, Leiden; 2 Department of Clinical Epidemiology, Leiden University Medical Center, Leiden; 3 Dutch Arthroplasty Register (LROI), ’s-Hertogenbosch; 4 Utrecht Institute for Pharmaceutical Sciences (UIPS), Division of Pharmacoepidemiology and Clinical Pharmacology, Utrecht University, Utrecht; 5 Department of Anesthesiology, Leiden University Medical Center, Leiden, The Netherlands

## Abstract

**Background and purpose:**

Concurrent benzodiazepine–opioid use is discouraged. We aimed to examine trajectories of benzodiazepine, opioid, and concurrent use following hip and knee arthroplasties for osteoarthritis (HA-OA, KA) and hip arthroplasty for fracture (HA-fracture).

**Methods:**

In this nationwide cohort study, the Dutch Arthroplasty Register (LROI) was linked to the Dutch Foundation for Pharmaceutical Statistics (SFK). We evaluated the proportion of patients with ≥ 1 medication dispensation in the year pre- and post-procedure and the prescribing physicians. Concurrent use was defined as ≥ 7 days overlap of benzodiazepine and opioid exposure.

**Results:**

We included 109,238 HA-OA, 17,464 HA fracture, and 113,306 KA. Between 2013 and 2021, the risk difference of postoperative benzodiazepine use was –7.2% (95% confidence interval [CI] –8.1 to –6.2%), while postoperative opioid use increased by 29.7% (CI 28.5–30.8%). Among HA-OA, from 2013 (4,391 arthroplasties) to 2021 (12,905 arthroplasties), the percentage of preoperative benzodiazepine users went from 18% to 13%, and postoperative from 23% to 14%. In contrast, preoperative opioid use changed from 25% to 33% and postoperative from 36% to 69%, In 2021, 6% of HA-OA, 11% of HA fracture, and 9% of KA received a concurrent dispensation in the first post-procedure year, predominantly in the first quarter. Orthopedic surgeons prescribed 29% (~18,732 prescriptions) of initial concurrent dispensations; subsequent prescriptions were mainly from general practitioners.

**Conclusion:**

From 2013–2022 in the Netherlands, benzodiazepine use decreased while opioid use increased among arthroplasty patients. Concurrent use remained frequent, despite safety recommendations against co-prescribing.

In Europe, over 3.1 million total hip arthroplasties are performed annually due to osteoarthritis (HA-OA) and fractures (HA fracture), along with 2.5 million total knee arthroplasties (KA) [1]. As the population ages, these procedures are increasingly performed on older, comorbid patients [2], highlighting the need to understand factors such as medication use that may impact patient outcomes.

In recent decades, opioid prescriptions surrounding orthopedic surgeries have risen dramatically [3,4]. Currently, approximately 75–90% of patients in European countries, such as the Netherlands, receive a post-arthroplasty opioid prescription [3]. Meanwhile, similar concerns have arisen regarding a comparable trend in the use of benzodiazepines across the general population [5]. This especially holds for elderly women [5], who represent a large part of the population undergoing arthroplasty [2].

Benzodiazepine use alone is associated with adverse effects such as cognitive impairment, falls, and respiratory depression [6]. Specifically, for arthroplasty patients, benzodiazepines increase the risk of perioperative fractures and the need for revision surgery [7,8]. These adverse effects become more frequent when benzodiazepines are used in combination with opioids [9], leading to a Black Box warning from the U.S. Food and Drug Administration (FDA) in 2017 against their concurrent use [10]. However, compared with opioids, there is limited data on the frequency of benzodiazepine prescriptions around arthroplasty procedures. Also, the frequency of the concurrent use of these medications, especially in light of rising opioid prescriptions, remains understudied.

To address this gap, we conducted a nationwide cohort study in the Netherlands to explore trends in opioid, benzodiazepine, and concurrent use around hip and knee arthroplasty between 2013 and 2022. Additionally, we investigated the timing of benzodiazepine, opioid, and concurrent use relative to surgery, as well as the medical specialties responsible for prescribing these medications. 

## Methods

### Study design

This is a nationwide cohort study, linking 2 databases: the Dutch Arthroplasty Register (LROI) and the Dutch Foundation for Pharmaceutical Statistics (SFK) register. The report was made according to the STROBE and RECORD guidelines.

### Data sources

The LROI covers all hospitals performing arthroplasties in the Netherlands. It contains procedure-related information and patient demographics at the time of the procedure. The level of completeness is > 98% for primary surgery [11].

The SFK contains pharmacy dispensation data from > 95% of the community pharmacies in the Netherlands [12]. The SFK dataset has been widely used in previous medication-use research in the Netherlands [13-15]. For the LROI procedures successfully matched to the SFK, the SFK provided individual-level pharmacy dispensation data for the year before and 2 years after the procedure. These data included the type of medication, the dispensation date, and the number of units dispensed.

A deterministic linkage between the LROI and the SFK was performed based on a combination of day of birth, sex, and 4-digit postal code. The detailed linkage procedure and its quality control, including the external validation procedure, can be found in Appendix A (Supplementary data) and a methodology paper with the same linkage strategy in a previous cohort has also been published [16].

### Study population

All primary HA-OA, HA fracture, and KA performed between 2013 and 2022 were included. Exclusion criteria were arthroplasties performed in patients < 18 years old and procedures unable to be linked to their pharmaceutical data. In addition, linked arthroplasties in which the retrieved pharmaceutical data did not contain prescriptions consistent with thromboprophylaxis (at least 1 prescription of low molecular weight heparin, direct oral anticoagulants, or warfarin between 30 days before or 35 days after the procedure) were excluded due to potential linkage unreliability (n = 105,099).

### Measures

The following patient and prosthesis information was retrieved from the LROI to characterize the population: age (years), sex (male/female), body mass index (BMI), current smoking status (yes/no), American Society of Anesthesiologists (ASA) classification, Charnley score, joint operated on (hip/knee), diagnosis (osteoarthritis/fracture), type of prosthesis (total, resurfacing, hemi-, unicondylar) and type of fixation (cemented, uncemented, hybrid).

Socioeconomic status (SES) scores based on the Statistics Netherlands score were extracted from the SFK. We extracted household‑level SES scores from the SFK based on the Statistics Netherlands (CBS) methodology. These scores combine (i) the national decile of welfare for each household, (ii) the highest educational level achieved by 1 household residents, and (iii) recent labor participation status (from continuously employed to retired). All scores were standardized against the overall Dutch population [17].

### Exposure definition

We selected the benzodiazepine and opioid dispensations according to the Anatomic-Therapeutic-Chemical (ATC) code: N05BA, N05CD, and N05CF for benzodiazepines and N02A for opioids. Duration of exposure for each prescription was calculated using the Individualized Dispensing Patterns (IDP) method, which calculates the time of exposition to each dispensation based on the number of units dispensed, a population estimate of duration per unit, and the number of days between previous dispensations of the same medication for each specific patient. This approach accounts for individual patterns of medication use and pro re nata prescriptions [18]. A concurrent dispensation was defined as an overlap in exposure ≥ 7 days between a benzodiazepine and an opioid dispensation, as has been previously used in articles exploring the association of concurrent use with outcomes [7]. A user was defined as a patient with ≥ 1 benzodiazepine, opioid, or concurrent medication dispensation in a period of interest.

### Data analysis

Data analyses and graphs were performed with R 4.3.1 (R Foundation for Statistical Computing, Vienna, Austria) and GraphPad Prism 8 software (https://www.graphpad.com/). Given their expected different clinical behavior, analyses were stratified by HA-OA, HA fracture, and KA. For descriptive statistics, continuous variables are shown as mean with standard deviation (SD), as the visual inspection revealed they were mostly normally distributed. Categorical variables are summarized as n (%).

First, we evaluated trends in medication use over the years. To do so, we estimated the annual proportion of benzodiazepine, opioid, and concurrent users in the year before or after the procedure between 2013 and 2022. As a summary measure, we calculated the absolute risk difference of being a user across all arthroplasties combined, along with the corresponding 95% confidence interval (CI), for the period 2013–2021. Among the benzodiazepine users, we evaluated the average number of dispensations and the average dispensed dose per patient, as had already been done for opioids [3]. Benzodiazepine dose was described in terms of the defined daily dose (DDD) and diazepam milligram equivalents (DME) per benzodiazepine user.

Second, we assessed the trajectories in medication use around the procedure moment. To do so, we evaluated the proportion of benzodiazepine, opioid, and concurrent users each 3-month period in the year before and 2 years after the surgery, accounting for patients who were no longer at risk (had died during follow-up). To account for potential changes in medication use over the years, we stratified these analyses by biennial groups of procedure years (2013–14, 2015–16, 2017–18, 2019–20, 2021–22). Among concurrent users, we evaluated per trimester which were the most frequent combinations of prescribers. Prescribers were stratified into general practitioner (GP), orthopedic surgeon, and others (e.g., rheumatologist and anesthesiologist). Because most patients who underwent surgery in 2022 had only a few months of follow-up at data extraction, these patients were excluded from the post-arthroplasty analyses.

Third, we evaluated whether the proportion of benzodiazepine, opioid, and concurrent users in the year before and after the procedure differed between clinically relevant groups: sex, age (below and above 75 years old), opioid-naive patients (no opioid use in the year before surgery) vs prevalent opioid users, and benzodiazepine-naive patients (no benzodiazepine use in the year before surgery) vs prevalent benzodiazepine users.

### Sensitivity analysis

To assess the impact of different definitions of concurrent use, we recalculated the proportion of patients with pre- and post-surgery concurrent use from 2013–2022 using stricter and looser definitions. For the looser definition, we modified the IDP method to use the 90th percentile of time per unit, prolonging the exposure time. For the stricter definition, we used the 50th percentile of time per unit, shortening the exposure time, and defined concurrent use as an overlap of ≥ 15 days.

### Ethics, funding, data sharing, use of AI tools, and disclosures

Approval by an ethics committee was waived by the non-Medical Research Involving Human Subjects Act (WMO in Dutch) committee of Division 1 of the Leiden University Medical Center (reference number: 2023-050).

This work was supported by the LROI (grant number RG 2023-002). The funders had no role in study design, analysis, or decision to publish.

The data on which this article was produced is not publicly available to preserve individuals’ privacy under the Dutch General Data Protection Regulation (AVG). Data is available from the authors upon reasonable request and with permission from the LROI and the SFK.

AI tools were used to make small additions and grammatical corrections to the text.

AD declares grants from NWO/NWA TAPTOE, ZonMW, US FDA, Grunenthal GmbH, AMO Pharma, Enalare, MSD, and Medtronic for research, educational, speaker, lecture, and consulting fees and study equipment. MB declares grants from NWO/NWA TAPTOE. All other authors have nothing to declare. Complete disclosure of interest forms according to ICMJE are available on the article page, doi: 10.2340/17453674.2025.44755

## Results

### Characteristics of the study population

**Figure 1:**
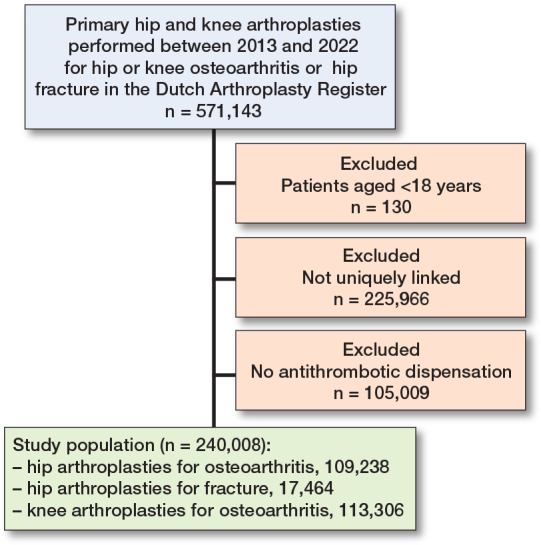
Flow diagram of the study population.

571,143 primary HA and KA of which 109,238 HA-OA, 17,464 HA fracture, and 113,306 KA were successfully linked with their medication data from the SFK (Figure 1). A comparison between the linked and unlinked population can be found in Supplementary Table 1.

The study population included 46%, 43%, and 50% of HA-OA, HA fracture, and KA, respectively, performed in men (Table 1). Mean age at procedure was 69, 78, and 68 years for HA-OA, HA fracture, and KA, respectively. Both OA groups were in the majority ASA II (69,714 [64%] HA-OA and 74,598 [66%] KA) while patients with HA fracture were most frequently ASA III (9,076, 52%). Mortality rates discriminated by type of procedure and procedure year were between 1 and 9 per 1,000 person-years in elective arthroplasty and between 22 and 146 per 1,000 person-years in patients operated on for hip fracture (Table 2).

**Table 1:** Characteristics of the study population. Values are count (%) or mean (SD)

	Hip arthroplasty		Knee arthroplasty
Characteristic	Osteoarthritis n = 109,238	Fracture n = 17,464	Osteoarthritis n = 113,306
Male sex	50,344 (46)	7,577 (43)	56,777 (50)
Age, mean (SD)	69 (10)	78 (10)	68 (9)
Age category			
< 65	31,371 (29)	1,736 (9.9)	38,615 (34)
65–69	20,530 (19)	1,779 (10)	22,765 (20)
70–74	23,211 (21)	2,534 (15)	23,468 (21)
75–79	18,883 (17)	3,066 (18)	17,421 (15)
80–84	10,959 (10)	3,507 (20)	8,554 (7.5)
85–89	3,659 (3.3)	2,940 (17)	2,250 (2.0)
≥ 90	625 (0.6)	1,902 (11)	233 (0.2)
Body mass index (SD) ^**a**^	27.5 (4.5)	24.8 (4.5)	29.5 (4.9)
Missing	2,751 (2.5)	1,329 (7.6)	2,734 (2.4)
Active smokers ^**a**^	10,671 (10)	1,639 (9.9)	9,381 (8.6)
Missing	4,636 (4.1)	888 (5.1)	4,550 (4.2)
Charnley classification			
A	46,062 (43)	––	46,028 (41)
B1	33,686 (31)	––	38,546 (34)
B2	24,308 (22)	––	23 120 (21)
C	2,653 (2.4)	–	2,945 (2.6)
Not applicable	332 (0.3)	––	283 (0.3)
Missing	970 (0.9)	––	802 (0.7)
ASA classification			
I	18,401 (17)	1,090 (6.3)	15,773 (14)
II	69,714 (64)	7,245 (42)	74,598 (66)
III–IV	21,005 (19)	9,076 (52)	22,803 (20)
Missing	132 (0.1)	53 (0.3)	118 (0.1)
Socioeconomic status			
score, mean (SD)	0.05 (0.22)	0.01 (0.22)	0.04 (0.22)
Missing	23,985 (22)	4,671 (27)	13,590 (12)
Type of prosthesis			
Total	109,238 (100)	5,492 (31)	95,869 (85)
Hemi/unicondylar	–	11,972 (69)	16,909 (15)
Fixation			
Cemented	23,231 (21)	11,333 (65)	96,587 (85)
Uncemented	75,124 (69)	4,938 (28)	12,970 (12)
Hybrid	10,883 (9.9)	1,142 (6.5)	3,673 (3.2)
Missing	100 (0.1)	51 (0.3)	76 (0.1)

ASA: American Society of Anesthesiologists Physical Status.

SD: standard deviation.

a Available since 2014.

**Table 2:** Benzodiazepine dispensation characteristics between 2013 and 2022

		Pre-arthroplasty					Post-arthroplasty			
Year	n	1-year mortality rate ^**a**^	BDZ users n (%)	Prescrip-tions per user, n	DDD per user	DME per user	BDZ users n (%)	Prescrip-tions per user, n	DDD per user	DME per user
Hip osteoarthritis										
2013	4,391	1.8	17.9	6.4	84	879	22.4	6.2	90	949
2014	8,169	5.3	17.0	6.6	83	886	20.9	6.3	87	926
2015	9,391	6.3	16.2	6.6	86	918	19.5	6.4	92	978
2016	10,079	5.7	15.7	6.4	80	859	18.5	6.0	84	902
2017	11,761	7.7	14.8	6.3	83	893	17.4	6.0	86	913
2018	12,735	8.7	14.0	6.4	79	850	16.3	5.9	82	880
2019	13,757	8.4	14.2	6.5	87	938	15.0	6.4	87	941
2020	10,859	9.3	14.2	6.1	89	966	14.6	6.2	90	975
2021	12,905	9.1	13.5	6.2	91	977	14.1	6.2	92	992
2022	15,191	–	12.5	6.4	89	972	––	–––	–––	–––
Hip fracture										
2013	322	21.9	20.8	10.9	90	1,003	27.0	13.8	106	1,132
2014	716	86.9	25.0	18.6	123	1,384	30.7	13.7	114	1,262
2015	1,105	92.1	23.2	13.2	97	1,017	30.1	10.9	92	988
2016	1,455	94.8	23.7	14.2	119	1,302	30.5	12.4	114	1,264
2017	1,916	149.1	20.9	13.7	93	1,002	29.1	10.4	91	991
2018	2,077	151.9	21.5	12.7	94	1,027	28.6	9.2	80	886
2019	2,255	148.0	20.0	14.0	121	1,313	25.6	9.6	105	1,140
2020	2,493	162.5	18.4	14.2	125	1,384	24.4	9.1	103	1,168
2021	2,527	146.3	18.4	12.7	113	1,232	25.7	9.0	101	1,100
2022	2,598	–	17.1	9.0	98	1,047	––	–––	––	––
Knee osteoarthritis										
2013	4,508	1.1	17.1	6.6	86	931	23.4	5.8	97	1,047
2014	8,417	3.2	16.3	7.0	86	917	22.0	6.3	96	1,015
2015	9,967	3.2	15.0	7.1	89	957	20.1	6.6	99	1,059
2016	10,677	3.8	14.5	7.3	92	989	20.0	6.2	102	1,084
2017	12,756	6.3	14.3	6.9	84	889	18.6	6.4	90	955
2018	13,513	6.1	13.1	6.6	83	890	17.5	5.7	91	973
2019	14,126	7.1	13.5	6.3	79	845	16.3	5.7	85	905
2020	11,272	8.6	13.5	6.6	84	899	16.3	6.0	89	950
2021	12,434	6.8	13.4	5.8	75	808	15.8	5.4	79	852
2022	15,636	–	11.3	6.1	81	868	–––	–––	–––	–––

BDZ: benzodiazepine, DDD: defined daily dose, DME: diazepam milligram equivalents. A user was defined as ≥ 1 dispensation in the year before or after procedure.

a per 1,000/year

### Medication use over time

**Figure 2:**
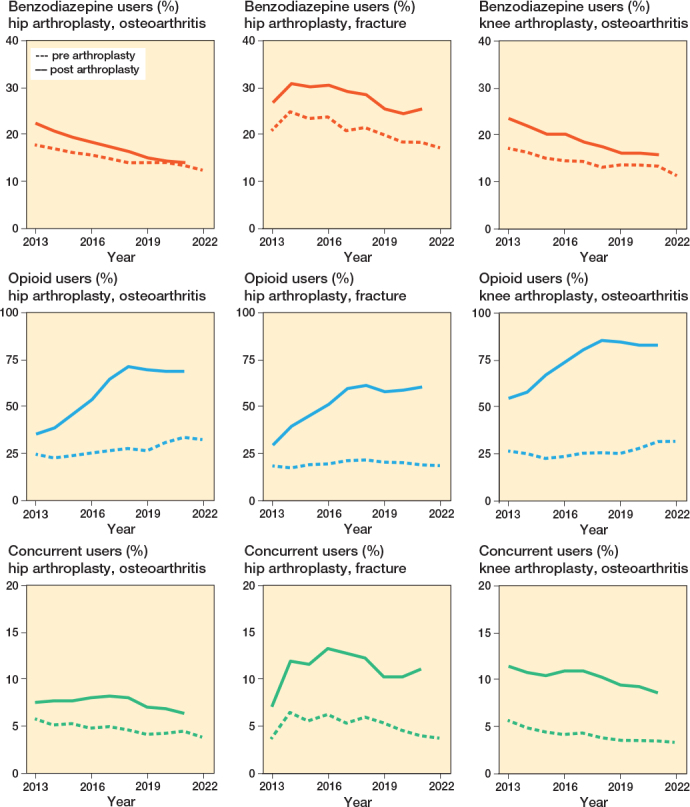
Benzodiazepine, opioid, and concurrent users between 2013 and 2022. A user was defined as ≥ 1 dispensation in the year before or after procedure. A concurrent dispensation was defined as an overlap ≥ 7 days between a benzodiazepine and an opioid dispensation.

Benzodiazepine use decreased over time in all groups both pre- and postoperatively (Figure 2, Supplementary Table 2). Between 2013 and 2021, the absolute risk difference across all arthroplasties was –3.7 (CI –4.6 to –2.8) for preoperative benzodiazepine use and –7.2 (CI –8.1 to –6.2) for postoperative use. In 2013, 18% (787/4,391) and 22% (984/4,391) of HA-OA patients were pre- and postoperative users, respectively, compared with 14% (1,748/12,905) and 14% (1,815/12,905) in 2021. HA fracture patients received more dispensations and higher doses on average than for other procedures (Table 2). In 2021, patients in the HA fracture group received fewer dispensations after arthroplasty compared with 2013, but the dispensed dose remained unchanged.

For opioids, on the contrary, the percentage of users in all groups increased steadily, peaking in 2018 and then stabilizing (see Figure 2, Supplementary Table 2). Between 2013 and 2021, the absolute risk difference across all arthroplasties was 6.4 (CI 5.3–7.4) for preoperative opioid use and 29.7 (CI 28.5–30.8) for postoperative use. Among HA-OA, the percentage of users in the year before surgery was 25% in 2013 (1,107/4,391), 28% in 2018 (3,611/12,735), and 34% in 2021 (4,416/12,905). In the year following surgery, the percentage of opioid users was 36% in 2013 (1,568/4,391), 72% in 2018 (9,135/12,735), and 69% in 2021 (8,898/12,905).

Concurrent use initially increased, peaking in 2017, and then showed a slight decline (see Figure 2, Supplementary Table 2). Between 2013 and 2021, the absolute risk difference across all arthroplasties was –0.9 (CI –1.4 to –0.3) for preoperative concurrent use and –1.0 (CI –1.7 to –0.2) for postoperative use. Among HA-OA patients, the percentage of concurrent users in the year before surgery was 6% in 2013 (276/4,391) and 5% in 2021 (718/12,905), while in the year after surgery, it was 8% in 2013 (373/4,391) and 6% in 2021 (982/12,905). Patients with HA fracture had the highest percentage of post-procedure concurrent users, reaching 11% in 2021 (400/2,527).

### Medication use around the arthroplasty procedure

**Figure 3:**
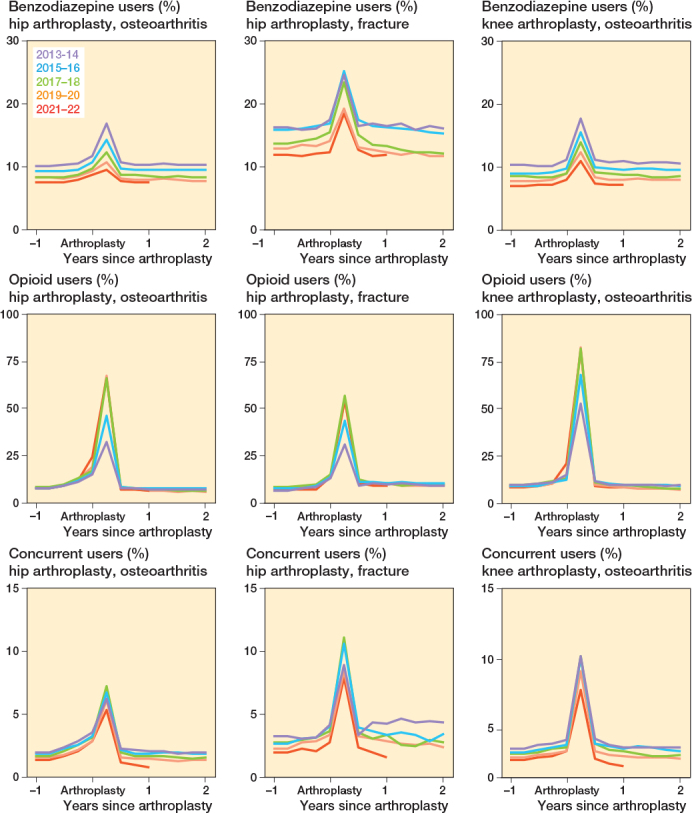
Benzodiazepine, opioid, and concurrent users before and after primary hip and knee arthroplasty. A user was defined as ≥ 1 dispensation in the trimester of interest. A concurrent dispensation was defined as an overlap ≥ 7 days between a benzodiazepine and an opioid dispensation.

For all procedures, the proportion of benzodiazepine, opioid, and concurrent users was highest in the first 3 months after surgery. During this period in 2021–2022, 10% of HA-OA (2674/28,096), 19% of HA fracture (951/5,125), and 11% of KA (3,070/28,070) patients were benzodiazepine users; 66% of HA-OA (18,535/28,096), 55% of HA fracture (2,820/5,125), and 82% of KA (22,874/28,070) patients were opioid users; and 6% of HA-OA (1,800/28,096), 11% of HA fracture (581/5,125), and 8% of KA (2,346/28,070) patients used both medications concurrently. For benzodiazepines, the percentage of users 2 years post-procedure was similar to that 1 year before surgery for all groups. However, in the HA fracture group, the proportion of opioid and concurrent users remained higher than pre-procedure levels for up to 2 years post-arthroplasty (Figure 3, Supplementary Tables 3–5).

At most times pre- and post-procedure, over 80% of concurrent prescriptions were by GPs (e.g., 26,965/32,712 prescriptions in the first preoperative quarter). The only exception was the first trimester post-arthroplasty (around 69,032 prescriptions), where about 27% of concurrent prescriptions involved an orthopedic surgeon (18,732).

### Medication use in clinically relevant subgroups

**Figure 4:**
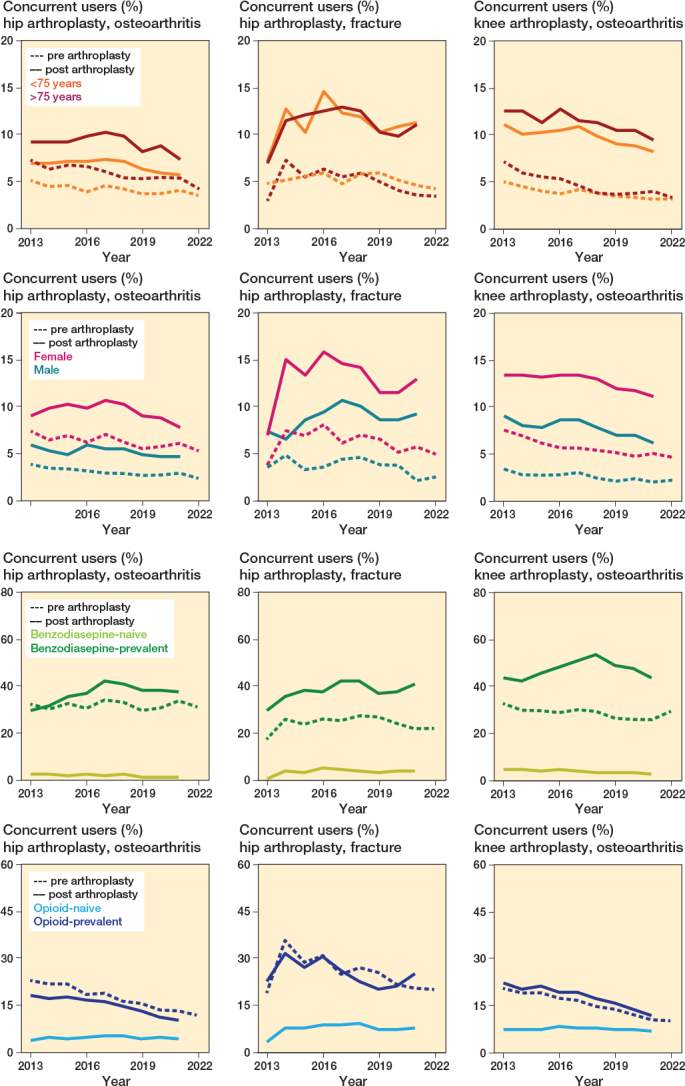
Stratified benzodiazepine and (concurrent) opioid users between 2013 and 2022. A user was defined as ≥ 1 concurrent benzodiazepine and opioid dispensation in the year before or after procedure. A concurrent dispensation was defined as an overlap ≥ 7 days between a benzodiazepine and an opioid dispensation.

In subgroup analyses, concurrent prescriptions after the procedure were highest among patients older than 75 years, in women and among prevalent users of either medication. For example, in HA-OA among prevalent benzodiazepine users, the percentage of post-surgery concurrent users in 2021 was 44% (767/1,748), compared with 2% (215/11,157) among benzodiazepine-naive patients (Figure 4).

### Sensitivity analyses

When changing the definition of concurrent prescription, the trends remained similar to those previously described. For example, in 2021 (12,905 HA-OA) according to the strict/loose definitions, 2%–6% of HA-OA (219–776) received a concurrent dispensation pre-procedure and between 2% and 10% post-surgery (194–1,268, Supplementary Table 6).

## Discussion

We aimed to examine trajectories of benzodiazepine, opioid, and concurrent use following HA-OA, KA, and HA fracture in the Netherlands. Between 2013 and 2022, the proportion of patients receiving benzodiazepines before or after HA or KA decreased. In contrast, opioid prescriptions increased substantially in both the pre- and postoperative periods. Concurrent prescribing of both medications was stable over time but remained frequent, particularly during the first 3 months after surgery and among patients who had undergone HA fracture. In that period, 29% of concurrent prescriptions involved orthopedic surgeons. Patients older than 75 years, women, and prevalent users of either medication received more concurrent prescriptions than their counterparts. The proportion of HA fracture patients who received an opioid or concurrent prescription remained higher than pre-procedure levels, even 2 years after arthroplasty.

Our results regarding the decrease in use of benzodiazepines align with reports from the general population in South Korea and Belgium [19,20], as well as global benzodiazepine sales data [21]. Additionally, since 2009, the Netherlands has implemented stricter regulations regarding the indications for which benzodiazepines are covered by health insurance [22], which may have further limited their use.

Regarding opioids, a previous article studying opioid use in the Netherlands after HA and KA up to 2018 showed a strong increase in their use [3], which was a trend also described among the general population in other studies [23,24]. Here, we updated the data through 2021 and found that opioid use after surgery has remained stable since 2018, remaining particularly frequent preoperatively and in the early postoperative period, which are also the periods when patients report more pain [25-27]. Importantly, we know from LROI publicly available data that the average pre- and postoperative reported pain has not changed since 2013 [25-27]. Therefore, the increase in opioid use is probably not related to changes in the pain experience of patients, but rather related to other factors, such as the reintroduction of oxycodone to postoperative pain management guidelines in 2013 [28], the growth in the use of fast-track discharge protocols with shorter lengths of stay [29], and the addition of postoperative pain relief during the first 72 hours after surgery as a benchmark for quality of care in the Netherlands since 2009 [30]. After the early postoperative period, patients with HA for osteoarthritis and KA returned to opioid use rates similar to those observed in the general population (~6%) [24]. However, because we lack information on the characteristics of these dispensations (e.g., duration, dose) in the general population, a fair comparison between the 2 groups cannot be made. For patients with HA fracture, the late postoperative opioid use prevalence remained higher than that of the general population.

The stability in post-procedure opioid use since 2018 contrasts with other studies in the general population from Australia and Canada [31,32], and in patients with HA and KA in the USA [33], where since 2017 post-procedure opioid use decreased. Many of these countries have introduced stricter regulations regarding opioid prescriptions in recent years [31] given their adverse effects [34], which has not been the case in the Netherlands. Decreasing post- procedure use is of importance, because opioid use is associated with long-term opioid misuse [35] and increased healthcare costs [36]. Furthermore, we found an increase in pre-procedure opioid use, which also is a major predictor of long-term opioid use after the arthroplasty procedure [37].

As for concurrent use, we found that the proportion of users increased between 2013 and 2017 but has since then slightly decreased. The trend aligns with reports from the USA [38], likely related to the FDA warning issued in 2017. Despite this tendency, concurrent benzodiazepine and opioid use remains frequent in the arthroplasty population, especially in the first trimester after the procedure and among patients with HA fracture, where it was around 11%. This is much higher than reports from the general population in the USA, at < 1% [38]. We also found that most concurrent prescriptions were issued by GPs. Studies from other countries have also shown that usually both medications are prescribed by the same provider [38]. This suggests that GPs are a critical point of intervention to reduce concurrent use of these medications.

### Strengths

A major strength of this study is the nationwide data on arthroplasty procedures and medication use over the past 10 years, providing an accurate picture of benzodiazepine and opioid use around arthroplasties in the Netherlands.

### Limitations

Both the SFK and LROI are data sets that were not specifically created to answer this research question, which generates some limitations. First, SFK is a dispensation register so we cannot verify that patients consumed the medication they were dispensed. Additionally, we were unable to link 58% of primary surgeries. Also, non‑linked cases were predominantly female, which could introduce bias given the known gender differences in medication use. Nevertheless, external validation against complete national data showed that our linked cohort remains broadly representative in terms of medication use of the overall arthroplasty population, suggesting that the influence of potentially introduced bias is minimal. Next, while our chosen period of at least 7 days to define concurrent use aligns with previous studies [39], we recognize that definitions vary and some authors have employed stricter criteria e.g. > 15 or > 30 days) [40,41]. However, as adverse effects of benzodiazepines and opioids most often manifest shortly after initiation [9,42,43], we consider our cutoff both reasonable and clinically relevant. Moreover, sensitivity analyses using a stricter exposure window produced similar dispensation trends, supporting the robustness of our findings. Finally, it could be argued that our results are only applicable to the Netherlands. However, the trends that we saw regarding opioid use align with previous reports from other European countries [44,45], and those regarding benzodiazepines also aligned with global dispensation trends [21]. This gives an indication that our results might be representative of a larger population.

### Conclusion

The proportion of benzodiazepine users among HA and KA patients declined to 13–18% preoperatively and 14–26% postoperatively, while opioid use increased markedly to 20–34% preoperatively and 61–83% postoperatively. Despite existing safety recommendations, 6–11% of patients used both medications concurrently after arthroplasty, most often during the first postoperative quarter.

*In perspective,* although general practitioners remained the primary prescribers of these combined dispensations, orthopedic surgeons accounted for one‑third of them in the first postoperative quarter. These findings indicate that opioid use remains a significant concern in peri-arthroplasty care and underscore the need for greater awareness of the potential risks associated with concurrent benzodiazepine use. There is a need for better communication between orthopedic surgeons and GP to reduce opioid use.

## Supplementary Material



## Supplementary data

Appendix A and Supplementary Table 1–6 are available as supplementary data on the article page, doi: 10.2340/17453674.2025.44755

## Notes

MYC and MG wrote the original draft. Conceptualization and funding acquisition was performed by RN, AD, and MG. LvS provided data curation for the LROI data. Formal analyses and visualization were performed by MYC. Methodology was done by MYC, RN, FR, MB, and MG. Supervision was done by RN, FR, MB, and MG. All authors interpreted the data and contributed to the final manuscript.

Handling co-editors: Marianne Westberg and Robin Christensen

*Acta* thanks Leiv Arne Rosseland and Claus Varnum for help with peer review of this manuscript.
