# Supplementary material for: 10-year trends in benzodiazepine, opioid, and concurrent use in hip and knee arthroplasty: a nationwide cohort study from the Netherlands
Source: Acta Orthop. 2025 Oct 10;96:771–9. doi: 10.2340/17453674.2025.44755 (PMC12512212; doi:10.2340/17453674.2025.44755)
Supplement: Supplementary file 1 [file ActaO-96-44755-s1.pdf]

Supplemental material

Appendix A. Quality control from the Linkage procedure between the Dutch Arthroplasty Register (LROI) and the Dutch Foundation for Pharmaceutical Statistics (SFK) register

Background

With the purpose of studying medication practices around hip and knee arthroplasty procedures, the Dutch Arthroplasty Register (LROI) [**1**] and the Dutch Foundation for Pharmaceutical Statistics (SFK) [**2**] register were merged in January 2024.

Merging was performed by a third trusted party using a deterministic linkage based on birthday, sex and patient 4-digit postal.

Methods

The quality checks were conducted in three stages, similar to how it was previously done for a previous LROI-SFK linkage in 2021 [**3**]. First, we evaluated the internal validity of the linkage. Second, we assessed the representativeness of the linked population compared to the source population. Finally, we externally validated the linkage.

All quality checks were performed for the overall linked population and specifically for the population with dispensations consistent with thromboprophylaxis, which is the clinical practice recommended after hip and knee arthroplasty by the Dutch guidelines [**4**]. Thromboprophylaxis was defined as at least one dispensing of LMWH, new oral anticoagulants (NOACs), or vitamin K antagonists (ATC codes: B01AA, B01AB, B01AE, and B01AF), in accordance with the Dutch clinical guidelines for thromboprophylaxis [**4**]. The guideline recommends thromboprophylaxis for 28–35 days after the procedure. Dispensations were assessed from 30 days before to 35 days after the procedure to capture both chronic users and patients given preoperative prescriptions. Because treatment duration can vary between centres, we did not filter by exact length. Thromboprophylaxis is intended for outpatient use, so nearly all hospitals either record prescriptions in their outpatient pharmacy or dispense medications directly through it—both of which report to the SFK, ensuring these dispensations are captured.

Internal validity

Five estimates of internal validity were calculated:

<u>Dispensations after death:</u> we received medication dispensing data from the SFK until 2 years after the procedure. Among the patients who died during that period according to the LROI, we evaluated whether they were dispensed any medications after death. We corrected for the fact that some outpatient pharmacies work in advance for repeated dispensations. If the number of units from the dispensing

could be divided by 7, the dispensing was provided maximum 3 weeks after death and was the last one for that patient, we assumed a repeated prescription.

Number of arthroplasties linked per SFK identifiers: we assessed whether any SFK identifier was linked to more than 4 primary arthroplasty procedures, since the maximum number of primary hip and knee arthroplasties that a person can receive is 4.

Patients correctly identified as having multiple arthroplasties: we identified patients who had multiple prostheses according to the LROI and evaluated the percentage in which these arthroplasties belonged to the same SFK identifier.

Antibiotics after revision surgery for infection: among the patients who had a minor revision surgery because of infection according to the LROI, we evaluated whether they were dispensed antibiotics (ATC code J01) according to the SFK after the revision procedure, as this is common clinical practice.

Thromboprophylaxis characteristics: for the patients with dispensations consistent with thrombo-prophylaxis, we obtained summary statistics of the type of medication, moment of dispensing, number of dispensations, and defined daily doses (DDD) dispensed to assess correspondence with current clinical practice in thromboprophylaxis.

Representativeness of the source population

As the LROI comprises all arthroplasties performed in the Netherlands, we compared the linked population to this source population to assess representativeness. We evaluated differences among linked and unliked patients in patient and arthroplasty-related characteristics.

External validation

In the linked primary arthroplasties, we calculated the proportion of arthroplasties with ≥1 opioid dispensing (ATC code N02A) in the year of their surgery, stratified by procedure year and joint (Hip or knee). These results were compared to the 2013–2018 estimates by Statistics Netherlands (CBS). The CBS holds information on all arthroplasties performed in the Netherlands, except those performed in private hospitals. It also holds medication reimbursement data which is linked to the procedure information by unique identifiers, therefore a near-perfect link is assured.

Results

In total, the LROI provided information on 571,143 primary arthroplasties, from which 345,045 (60.4%) could be linked to the SFK. From these arthroplasties, 240,018 (42.0%) had a prescription consistent with thromboprophylaxis.

Internal validity

Dispensing after death: During the study period, a total of 13,637 patients died. The percentage of arthroplasties with dispensations after death was 9.8% (10.9% for those with thromboprophylaxis) as shown in Table A.1. When adjusting for possible repeat prescriptions, the percentage of dispensations after death was 4.6% (4.3% for those with thromboprophylaxis).

Number of arthroplasties linked per SFK identifiers: 1 SFK identifier was linked to more than 4 primary procedures (<1%). Among the patients with thromboprophylaxis no patients were linked to more than 4 primary arthroplasties (Table A.1).

Patients correctly identified as having multiple arthroplasties: according to the LROI, 47.118 patients received multiple primary arthroplasties. From these patients, 46,695 (99.1%) were identified as being the same individual by the SFK (99.7% for those with thromboprophylaxis, Table A.1).

Antibiotics after revision surgery for infection: during the study period, 1,755 primary procedures received a minor revision arthroplasty due to infection. From these, 82.5% received an antibiotic dispensing after the revision procedure (88.6% for those with thromboprophylaxis, Table A.1).

Thromboprophylaxis characteristics: among arthroplasties with dispensations consistent with thromboprophylaxis, the most frequently dispensed medication was LMWH (75.6%), the median moment of dispensing was 2 days after the procedure and most patients received 1 dispensing with a median DDD per dispensing of 28. These indicators were in line with the current recommendations for thromboprophylaxis in the Dutch clinical guidelines.

Table A.1. Linkage quality checks of primary procedures. Values are count (%)

	**Overall**	**With thrombo-prophylaxis** [a]
	345,045 (60)	240,018 (42)
**Dispensing after death**		
Deaths within the study period, n	13,637	6,355
Arthroplasties with dispensing after death, n (%)	1,341 (9.8)	694 (10.9%)
Corrected arthroplasties with dispensing after death, n (%)	634 (4.6)	271 (4.3)
**Arthroplasties per patient**		
SFK identifier linked to >4 primary arthroplasties, [b] (%)	<0.1	<0.1
**Identification of patients with multiple procedures**		
Patients with multiple primary procedures according to LROI	47,118	29,929
Patients identified as the same individual by the SFK, n [b] (%)	46,695 (99.1)	29,850 (99.7)
**Antibiotics after revision for infection**		
Partial revision for infection within the study period	1,755	1 250
Antibiotic dispensing after the procedure, n (%)	1,447 (82.5)	1,107 (88.6)

[a] Arthroplasties with at least one dispensing of LMWH, NOACS or vitamin K antagonist prescribed between 30 days before surgery and 35 days after.
n = number of arthroplasties,
n [b] = number of persons.

Representatives of the linked population

When comparing the linked arthroplasties with thromboprophylaxis to the unlinked/no thromboprophylaxis procedures, they were more frequently performed in males (47.8% vs 27.3%), more frequently knee interventions (47.2% vs 43.9%) and less frequently hemiarthroplasties (5.0% vs 10.8%). For the other patient and surgery-related characteristics the two populations were quite comparable (Table A.2).

External validation

According to CBS data, the percentage of patients with an opioid prescription in the year of a primary hip arthroplasty ranged from 39.8% to 67.5%, and for knee arthroplasty, it ranged from 57.3% to 85.9% (2013-2018). From the LROI-SFK linkage data, this proportion was 28.1% to 63.3% for hip arthroplasty and 41.7% to 78.8% for knee arthroplasty. The average absolute percentage difference between CBS and LROI-SFK data was 11.5%. For primary arthroplasties with thromboprophylaxis, the prevalence of opioid prescription was 41.1% to 74.7% after hip arthroplasty and 57.7% to 88.2% after knee arthroplasty, with an average absolute percentage difference of 1.4% (Figure A.1).

Table A.2. The linked primary arthroplasty population compared with the not-linked population
Values are count (%) or mean (standard deviation [SD])

	**Linked with thrombo-prophylaxis** [a]	**Not linked/ No thromboprophylaxis**
	n = 240,018	n = 331,125
Sex, male	114,700 (47.8)	90,429 (27.3)
Age, years, mean (SD)	69.4 (9.6)	70.7 (10.3)
Body mass index [b], mean (SD)	28.2 (4.9)	28.1 (5.2)
Smokers [b]	21,691 (9.4)	26,929 (8.7)
Joint, knee	113,306 (47.2)	145,506 (43.9)
Osteoarthritis	222,554 (92.7)	286,966 (86.7)
Type of arthroplasty		
Hemiarthroplasty	11,972 (5.0)	35,646 (10.8)
Patellofemoral knee	504 (0.2)	818 (0.2)
Total hip	114,740 (47.8)	149,973 (45.3)
Total knee	95,869 (39.9)	123,918 (37.4)
Unicondylar knee	16,909 (7.0)	20,736 (6.3)
Charnley classification [b]		
A	95,571 (39.8)	127,264 (38.4)
B1	72,818 (30.3)	90,142 (27.2)
B2	48,108 (20.0)	64,359 (19.4)
C	5,860 (2.4)	9,147 (2.8)
Not applicable	12,750 (5.3)	29,610 (8.9)
ASA classification		
ASA I	35,270 (14.7)	44,283 (13.4)
ASA II	151,560 (63.1)	203,790 (61.7)
ASA III–IV	52,885 (22.0)	82,397 (24.9)
Socioeconomic status, deciles [b]	5.10 (2.85)	5.12 (2.81)
**Preoperative PROMS** [b], mean (SD)		
EQ-5D VAS	66.8 (20.2)	65.4 (20.7)
NRS pain during activity	7.2 (2.0)	7.3 (2.0)

	Linked with thrombo-prophylaxis [a]	Not linked/ No thromboprophylaxis
	n = 240,018	n = 331,125
NRS pain in rest	5.1 (2.6)	5.3 (2.6)
HOOS-PS	48.1 (17.5)	49.6 (18.1)
OHS	23.2 (8.6)	22.4 (8.7)
KOOS-PS	50.0 (15.0)	51.8 (15.3)
OKS	23.8 (7.7)	22.7 (7.7)

[a] Arthroplasties with at least one dispensing of LMWH, NOACS or vitamin K antagonist prescribed between 30 days before surgery and 35 days after.
[b] Available since 2014.
ASA = American Society of Anesthesiologists Physical Status, PROMs = Patient-reported outcomes measurement, NRS = Numeric Rating Scale for pain, HOOS-PS = Hip disability and Osteoarthritis Outcome Score – Physical function Short form, OHS = Oxford Hip Score, KOOS-PS = Knee injury and Osteoarthritis Outcome Score – Physical function Short form, OKS = Oxford Knee Score.
Score.

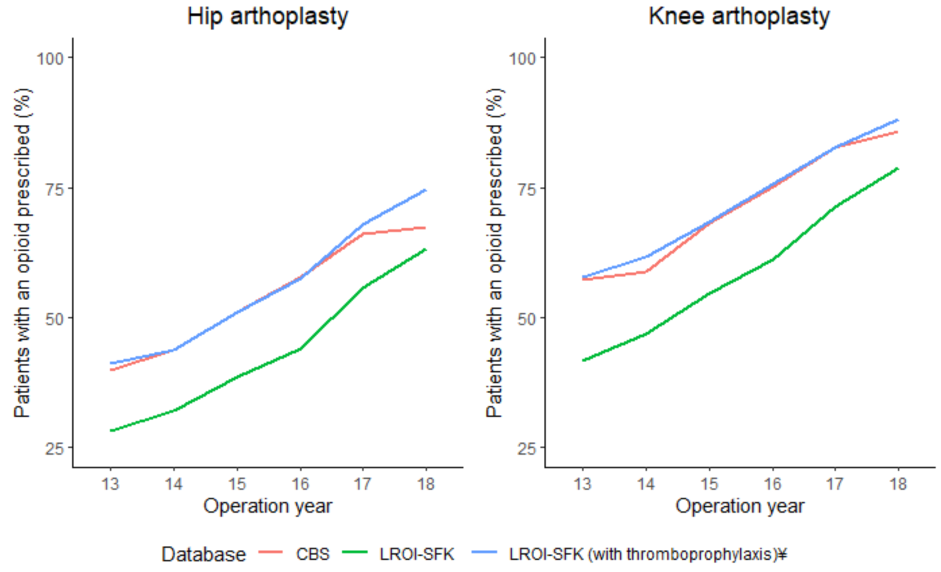

Figure A.1. Annual percentage of prescribed opioids in primary arthoplasties according to source of information. ¥Arthoplasties with at least one dispensing of LMWH, NOACS or vitamin K antagonist prescribed between 30 days before surgery and 35 days after

Conclusion

In general, the 2024 linkage based on birthdate, sex, and 4-digit postcode achieved a high percentage of linked arthroplasties and performed well in quality checks for internal validity. When considering only patients with prescriptions consistent with thromboprophylaxis, internal validity improved, and opioid prescription prevalence matched what has been found in Dutch registries linked by unique identifiers. Therefore, uniquely linked primary arthroplasties with at least one dispensing of thromboprophylaxis were considered as the study population. Of notice, these arthroplasties were less common in male patients and less frequently hemiarthroplasties, indicating a potentially healthier population, which needs to be considered when interpreting future results.

References

1. Dutch Arthroplasty Register (LROI). Wat is de LROI? 2023. Available from https://www.lroi.nl/over-de-lroi/wat-is-de-lroi/ (accessed 12-12-2023 2023)
2. Dutch Foundation for Pharmaceutical Statistics (SFK). *Data en feiten.* Den Haag, Netherlands, 2023
3. van Brug HE, Rosendaal FR, van Steenbergen LN, Nelissen R, Gademan MGJ. Data linkage of two national databases: Lessons learned from linking the Dutch Arthroplasty Register with the Dutch Foundation for Pharmaceutical Statistics. *PLoS One* 2023; 18: e0282519
4. Dutch Federation of Medical Specialists (FMS). Antitrombotisch beleid. 2020. Available from https://richtlijnendatabase.nl/richtlijn/antitrombotisch_beleid/preventie_vte/keuze_en_duur_profylaxe_grote_ingrepen.html (accessed 01-09-2025).

Supplemental Table 1. The primary arthroplasty population linked compared with the not-linked population. Values are count (%) or mean (standard deviation [SD])

	**Not-linked arthroplasties**	**Linked arthroplasties**	**SMD**
	n = 331,005	n = 240,008	
Sex male	90,372 (27.3)	114,700 (47.8)	
Age, years, mean (SD)	71 (10)	69 (10)	0.13
Body mass index [a], mean (SD)	28.1 (5.2)	28.2 (4.9)	0.03
missing	16,854 (5.1)	6,814 (2.8)	
Active smokers [a]	26,919 (8.7)	21,691 (9.4)	
missing	23,353 (7.1)	10,074 (4.2)	
Joint, knee	145,465 (43.9)	113,306 (47.2)	
Osteoarthritis	286,860 (86.7)	222,554 (92.7)	
Type of arthroplasty			
Hemi hip arthroplasty	35,637 (10.8)	11,972 (5.0)	
Total hip arthroplasty	149,903 (45.3)	114,740 (47.8)	
Total knee arthroplasty	123,888 (37.4)	95,869 (39.9)	
Other	21,588 (6.5)	17,437 (7.3)	
Charnley classification			
A	127,207 (38.9)	95,571 (39.8)	
B1	90,113 (27.5)	72,818 (30.3)	
B2	64,345 (19.7)	48,108 (20.0)	
C	9,141 (2.8)	5,860 (2.4)	
Not applicable	29,610 (9.0)	12,750 (5.3)	
missing	10,601 (3.2)	4,911 (2.0)	
ASA classification			
I	44,263 (13.4)	35,270 (14.7)	
II	203,721 (61.7)	151,560 (63.1)	
III–IV	82,366 (24.9)	52,885 (22.0)	
missing	655 (0.2)	303 (0.1)	
Socioeconomic status deciles	5.1 (2.8)	5.1 (2.9)	0.004
missing	2,312 (0.7)	1,204 (0.5)	

[a] Available since 2014.

SMD = standardized mean difference, ASA = American Society of Anesthesiologists Physical Status.

Supplemental Table 2. Benzodiazepine, opioid and concurrent users between 2013 and 2022

Year	n	Benzodiazepines users (%) [a]		Opioids users (%) [a]		Concurrent users (%) [a]	
		Pre arthroplasty	Post arthroplasty	Pre arthroplasty	Post arthroplasty	Pre arthroplasty	Post arthroplasty
**Hip arthroplasty (osteoarthritis)**							
2013	4,391	17.9	22.4	25.2	35.7	5.9	7.7
2014	8,169	17.0	20.9	23.6	39.3	5.2	7.8
2015	9,391	16.2	19.5	24.5	46.8	5.4	7.8
2016	10,079	15.7	18.5	25.9	54.5	4.8	8.1
2017	11,761	14.8	17.4	27.1	65.3	5.1	8.3
2018	12,735	14.0	16.3	28.4	71.7	4.7	8.1
2019	13,757	14.2	15.0	27.1	69.9	4.3	7.0
2020	10,859	14.2	14.6	31.7	69.4	4.4	6.9
2021	12,905	13.5	14.1	34.2	69.0	4.6	6.4
2022	15,191	12.5	–	32.9	–	3.9	–
**Hip arthroplasty (fracture)**							
2013	322	20.8	27.0	19.3	30.4	3.7	7.1
2014	716	25.0	30.7	18.2	40.4	6.6	12.0
2015	1,105	23.2	30.1	19.5	45.8	5.6	11.6
2016	1,455	23.7	30.5	20.3	51.7	6.3	13.3
2017	1,916	20.9	29.1	21.8	59.9	5.4	12.8
2018	2,077	21.5	28.6	22.1	61.9	6.0	12.3
2019	2,255	20.0	25.6	21.0	58.1	5.4	10.3
2020	2,493	18.4	24.4	20.8	59.5	4.6	10.2
2021	2,527	18.4	25.7	19.7	60.8	4.1	11.2
2022	2,598	17.1	–	19.1	–	3.8	–
**Knee arthroplasty (osteoarthritis)**							
2013	4,508	17.1	23.4	26.9	55.1	5.6	11.4
2014	8,417	16.3	22.0	26.0	58.7	4.9	10.8
2015	9,967	15.0	20.1	23.2	68.0	4.5	10.5
2016	10,677	14.5	20.0	24.3	74.5	4.2	11.0
2017	12,756	14.3	18.6	26.0	81.1	4.4	11.0
2018	13,513	13.1	17.5	26.3	86.2	3.9	10.3
2019	14,126	13.5	16.3	26.0	84.9	3.6	9.4
2020	11,272	13.5	16.3	28.9	83.8	3.6	9.3
2021	12,434	13.4	15.8	32.6	83.4	3.5	8.5
2022	15,636	11.3	–	32.0	–	3.4	–

[a] A user was defined as receiving ≥1 dispensation in the year before or after procedure. A concurrent dispensation was defined as an overlap ≥7 days between a benzodiazepine an opioid dispensation.

Supplemental Table 3. Benzodiazepine users (%) before and after primary hip and knee arthroplasty.

	**Hip arthroplasty (osteoarthritis)**					**Hip arthroplasty (fracture)**					**Knee arthroplasty (osteoarthritis)**				
Year	2013–14	2015–16	2017–18	2019–20	2021–22	2013–14	2015–16	2017–18	2019–20	2021–22	2013–14	2015–16	2017–18	2019–20	2021–22
n	12,560	19,470	24,496	24,616	28,096	1,038	2,560	3,993	4,748	5,125	12,925	20,644	26,269	25,398	28,070
**Moment (months)** [a]															
–12 to –9	10.2	9.3	8.4	8.3	7.6	16.4	15.9	13.7	12.9	12.0	10.3	9.0	8.5	7.8	7.1
–9 to –6	10.3	9.3	8.5	8.1	7.6	16.0	16.1	14.1	13.6	11.8	10.2	9.1	8.4	7.9	7.1
–6 to –3	10.5	9.6	8.8	8.5	7.9	16.2	16.4	14.6	13.3	12.2	10.3	9.1	8.5	7.9	7.1
–3 to arthroplasty	11.8	10.8	9.7	9.5	8.7	17.5	17.0	15.5	14.0	12.4	11.1	9.8	8.9	9.0	8.0
Arthroplasty to 3	16.8	14.4	12.3	10.8	9.5	24.6	25.2	23.5	19.4	18.6	17.7	15.5	13.9	12.3	10.9
3 to 6	10.7	9.8	8.7	8.2	7.8	16.5	17.5	15.1	13.1	12.7	11.1	9.9	9.3	8.4	7.4
6 to 9	10.5	9.6	8.8	8.1	7.6	16.9	16.5	13.6	12.7	11.7	10.8	9.8	8.9	7.9	7.2
9 to 12	10.5	9.6	8.6	8.1	7.5	16.6	16.2	13.4	12.4	12.0	10.9	9.7	8.8	8.0	7.2
12 to 15	10.5	9.6	8.5	8.1	–	16.9	16.2	12.7	12.0	–	10.5	9.7	8.7	8.1	–
15 to 18	10.4	9.5	8.5	8.1	–	16.0	15.9	12.4	12.3	–	10.7	9.8	8.4	8.1	–
18 to 21	10.3	9.5	8.4	7.8	–	16.5	15.4	12.3	11.7	–	10.7	9.5	8.5	7.9	–
21 to 24	10.4	9.6	8.3	7.8	–	16.2	15.4	12.1	11.8	–	10.6	9.6	8.5	7.9	–

[a] In relation to the moment of the arthroplasty. A user was defined as receiving ≥1 dispensation in the year before or after procedure.

Supplemental Table 4. Opioid users (%) before and after primary hip and knee arthroplasty.

	Hip arthroplasty (osteoarthritis)					Hip arthroplasty (fracture)					Knee arthroplasty (osteoarthritis)				
Year	2013–14	2015–16	2017–18	2019–20	2021–22	2013–14	2015–16	2017–18	2019–20	2021–22	2013–14	2015–16	2017–18	2019–20	2021–22
n	12,560	19,470	24,496	24,616	28,096	1,038	2,560	3,993	4,748	5,125	12,925	20,644	26,269	25,398	28,070
**Moment (months)** [a]															
–12 to –9	7.9	8.1	8.6	7.9	7.6	6.7	7.9	8.8	8.4	7.0	9.9	9.2	9.8	9.3	8.5
–9 to –6	9.0	9.3	9.7	9.2	9.0	7.6	7.9	9.2	8.4	7.5	10.2	9.5	10.5	9.4	9.1
–6 to –3	11.3	11.9	13.0	12.2	11.6	8.3	8.9	10.1	9.3	7.5	11.3	11.4	11.8	10.5	10.2
–3 to arthroplasty	15.4	15.7	17.4	18.9	24.5	13.1	13.6	14.8	14.6	13.4	14.9	12.4	12.8	14.7	20.9
Arthroplasty to 3	32.4	46.4	65.8	67.0	66.0	30.9	43.4	56.9	54.3	55.0	53.0	68.0	81.9	82.8	81.5
3 to 6	7.7	8.2	8.6	7.1	7.1	9.5	11.3	12.5	11.0	10.7	11.7	11.9	11.5	9.5	9.4
6 to 9	7.6	7.7	7.6	7.1	6.9	10.2	11.4	10.5	10.2	9.0	10.0	10.3	9.8	8.9	8.5
9 to 12	7.3	7.7	7.3	6.5	6.6	9.6	10.2	10.4	10.2	8.9	9.5	10.2	9.6	8.3	8.2
12 to 15	7.3	8.0	7.3	6.4	−	10.2	11.2	9.3	9.2	−	9.9	10.1	9.1	8.2	−
15 to 18	7.0	7.7	6.6	6.2	−	9.9	10.5	9.8	9.4	−	9.8	9.7	8.4	7.9	−
18 to 21	7.0	7.6	6.3	6.2	−	9.2	10.2	10.0	9.3	−	9.3	9.7	8.0	7.7	−
21 to 24	6.9	7.5	6.3	6.1	−	9.4	10.4	9.7	9.2	−	9.6	9.3	7.6	7.4	−

[a] In relation to the moment of the arthroplasty. A user was defined as receiving ≥1 dispensation in the year before or after procedure.

Supplemental Table 5. Concurrent benzodiazepine and opioid users (%) before and after primary hip and knee arthroplasty.

	**Hip arthroplasty (osteoarthritis)**					**Hip arthroplasty (fracture)**					**Knee arthroplasty (osteoarthritis)**				
Year	2013–14	2015–16	2017–18	2019–20	2021–22	2013–14	2015–16	2017–18	2019–20	2021–22	2013–14	2015–16	2017–18	2019–20	2021–22
n	12,560	19,470	24,496	24,616	28,096	1,038	2,560	3,993	4,748	5,125	12,925	20,644	26,269	25,398	28,070
**Moment (months)** [a]															
–12 to –9	1.9	1.7	1.5	1.4	1.3	3.2	2.6	2.7	2.2	1.9	2.2	1.9	1.7	1.5	1.2
–9 to –6	2.3	2.2	2.0	1.7	1.5	3.0	2.9	2.9	2.6	2.2	2.4	2.0	1.9	1.6	1.4
–6 to –3	2.8	2.5	2.4	2.1	2.0	3.1	3.1	3.1	2.8	2.0	2.5	2.3	2.1	1.8	1.5
–3 to arthroplasty	3.5	3.2	3.1	2.7	2.7	4.0	3.9	3.5	3.3	2.7	2.9	2.5	2.3	1.9	2.0
Arthroplasty to 3	6.2	6.7	7.1	6.1	5.2	8.9	10.5	11.0	8.3	7.9	9.5	9.5	9.6	8.4	6.8
3 to 6	2.1	2.0	1.8	1.4	1.0	3.3	3.9	3.5	3.2	2.3	2.9	2.6	2.5	1.9	1.3
6 to 9	2.1	1.8	1.6	1.3	0.9	4.2	3.6	3.0	3.0	1.9	2.4	2.3	2.0	1.7	1.0
9 to 12	1.9	1.8	1.5	1.3	0.6	4.2	3.2	3.2	2.7	1.4	2.2	2.1	1.9	1.5	0.7
12 to 15	1.9	1.8	1.5	1.3	–	4.5	3.4	2.4	2.5	–	2.3	2.3	1.7	1.5	–
15 to 18	1.8	1.8	1.4	1.2	–	4.3	3.2	2.3	2.4	–	2.2	2.2	1.6	1.4	–
18 to 21	1.8	1.7	1.4	1.3	–	4.3	2.8	2.9	2.6	–	2.3	2.1	1.5	1.4	–
21 to 24	1.8	1.7	1.4	1.3	–	4.3	3.4	2.6	2.3	–	2.3	2.0	1.6	1.4	–

[¥]In relation to the moment of the arthroplasty. A user was defined as receiving ≥1 dispensation in the year before or after procedure. A concurrent dispensation was defined as an overlap ≥7 days between a benzodiazepine an opioid dispensation.

Supplemental Table 6. Worst case scenario simulation of the concurrent benzodiazepine and opioid use prevalence in patients before and after primary hip and knee arthroplasty

Year	n	Concurrent users according to loose criteria (%) [a]		Concurrent users according to strict criteria (%) [b]	
		Pre arthroplasty	Post arthroplasty	Pre arthroplasty	Post arthroplasty
**Hip arthroplasty (osteoarthritis)**					
2013	4,391	6.8	11.8	3.1	3.4
2014	8,169	5.9	11.3	2.4	3.1
2015	9,391	6.1	11.8	2.3	3.0
2016	10,079	5.8	12.3	2.1	2.7
2017	11,761	6.0	12.7	2.1	2.8
2018	12,735	5.7	12.3	1.8	2.4
2019	13,757	5.2	11.3	1.8	1.9
2020	10,859	5.9	10.9	1.8	1.8
2021	12,905	6.0	9.8	1.7	1.5
2022	15,191	4.7	–	1.4	–
**Hip arthroplasty (fracture)**					
2013	322	5.9	14.6	1.9	3.7
2014	716	8.9	19.8	3.1	5.4
2015	1,105	7.0	19.4	2.2	3.1
2016	1,455	7.1	21.0	2.5	4.5
2017	1,916	6.2	22.5	1.8	3.8
2018	2,077	7.1	21.6	2.6	3.4
2019	2,255	6.3	18.9	1.9	2.4
2020	2,493	5.3	18.6	1.7	2.9
2021	2,527	5.4	19.3	1.8	2.8
2022	2,598	4.9	–	1.5	–
**Knee arthroplasty (osteoarthritis)**					
2013	4,508	6.4	15.6	2.7	4.5
2014	8,417	5.7	15.4	2.4	4.2
2015	9,967	5.2	15.0	2.1	3.8
2016	10,677	4.9	15.8	1.9	3.7
2017	12,756	5.1	16.0	2.0	3.6
2018	13,513	4.6	15.1	1.7	3.1
2019	14,126	4.7	14.1	1.5	2.8
2020	11,272	5.3	14.0	1.4	2.5
2021	12,434	5.4	13.2	1.3	2.2
2022	15,636	4.2	–	1.2	–

[a] 90th percentile as the population estimate of time per unit.

[b] 50th percentile as the population estimate of time per unit and an overlap of exposure ≥14 days.
